# Brain Metabolic Activity Measured by [^18^F]FDG PET/CT Predicts Survival in Patients with Advanced Non–Small Cell Lung Cancer

**DOI:** 10.2967/jnumed.125.271400

**Published:** 2026-07

**Authors:** Julie Auriac, Ghada Lemoudda, Narinée Hovhannisyan-Baghdasarian, Manuel Pires, Lalith Kumar Shiyam Sundar, Paulette Salamoun-Feghali, Romain-David Seban, Nina Jehanno, Christophe Nioche, Marie Luporsi, Thomas Beyer, Alain Livartowski, Nicolas Girard, Irène Buvat, Fanny Orlhac

**Affiliations:** 1 Institut Curie, PSL, Université Versailles Saint-Quentin, Inserm, CNRS, Université Paris-Saclay, IRIS, Orsay, France;; 2 Quantitative Imaging and Medical Physics Team, Medical University of Vienna, Vienna, Austria;; 3 DIGIT-X Lab, Department of Radiology, LMU University Hospital, Munich, Germany;; 4 Institut Curie, Institut du Thorax Curie-Montsouris, Paris, France; and; 5 Department of Nuclear Medicine, Institut Curie, Saint-Cloud, Paris, France

**Keywords:** 18F-FDG PET/CT, non–small cell lung cancer, radiomics, survival

## Abstract

[^18^F]FDG PET/CT images play a key role in the management of patients with non–small cell lung cancer (NSCLC). In these scans, the focus is on detected tumors and their characteristics, neglecting information from other organs or tissues. We investigated whether the mean brain [^18^F]FDG uptake (brain SUV_mean_) is associated with overall survival (OS) in patients with advanced NSCLC. **Methods:** This retrospective study included patients with advanced NSCLC who underwent pretreatment [^18^F]FDG PET/CT scans between 2010 and 2023. Clinical and biologic data, tumor radiomic features, and brain SUV_mean_ were collected. The ability of these features to predict OS was evaluated using univariable and multivariable Cox regression models. The correlation between brain SUV_mean_ and clinical, imaging, and blood biomarkers was investigated using Spearman correlation coefficients. **Results:** Patients were chronologically divided into a discovery set (*n* = 234; mean age, 64 ± 11 y) and test set (*n* = 146; mean age, 66 ± 11 y). In the discovery set, univariable analysis showed that high brain SUV_mean_ (greater than or equal to the median) was associated with longer OS (hazard ratio [HR], 0.83; 95% CI, 0.76–0.92; *P* < 0.001). Brain SUV_mean_ was significantly lower in patients who died within 1 y compared with those who were still alive at the same time point (median brain SUV_mean_, 4.9 ± 1.4 vs. 5.7 ± 1.5, respectively; *P* < 0.001). Multivariable analysis revealed that brain SUV_mean_ was an independent prognostic factor for OS (HR, 0.89; 95% CI, 0.80–0.98; *P* = 0.02), which was confirmed in the test set (*P* < 0.001). Brain SUV_mean_ was independent of the radiomic features quantifying tumor involvement (*r* < 0.24, *n* = 380) and significantly correlated but complementary to several blood biomarkers including C-reactive protein (*r* = −0.37, *n* = 110 patients). The prognostic significance of brain SUV_mean_ persisted in patients without brain metastases (*P* < 0.001). **Conclusion:** Low brain metabolic activity was associated with increased mortality in patients with advanced NSCLC. Brain SUV_mean_ was an independent prognostic factor that may aid in patient stratification, although its interpretation requires further investigation.

## Visual Abstract

**Figure:**
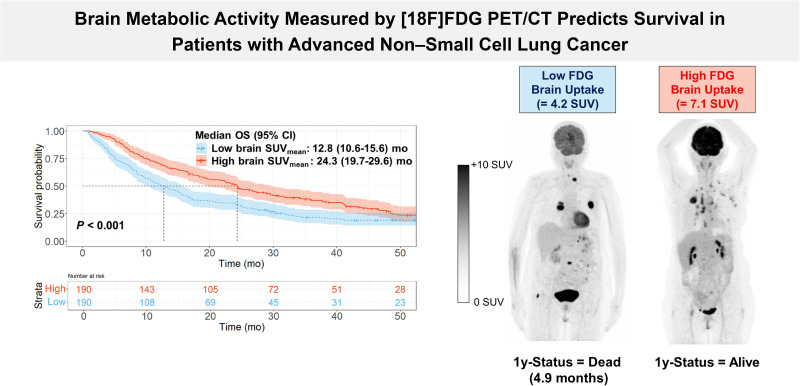

Lung cancer was the leading cause of cancer-related deaths worldwide in 2022, with a total of 1.8 million deaths (18%) (1). Despite promising new therapies, the prognosis for patients is relatively poor, with overall survival (OS) ranging from 37.5 mo in early-stage disease to 4.8 mo in the metastatic stage (2). In clinical practice, the management of metastatic lung cancer remains challenging, as some patients are resistant to treatments or experience only short-term benefits without any significant impact on their survival (3,4).

Numerous studies have shown that advanced analysis of whole-body [^18^F]FDG PET/CT images could provide valuable information regarding tumor invasion to predict survival in patients with lung cancer. Total metabolic tumor volume (TMTV), total lesion glycolysis (TLG), SUV_max_, and the maximum distance between 2 lesions (D_max_) are PET-derived biomarkers reflecting metabolic tumor burden, tumor activity, and disease dissemination and have a strong prognostic value for patient survival (5–7).

Recent studies have suggested that metabolic activity and density in various organs and tissues measured from whole-body [^18^F]FDG PET/CT images also provide valuable information for predicting patient outcomes in lung cancer by reflecting the patient’s general condition (8,9). For example, [^18^F]FDG uptake measured in lymphoid organs may reflect systemic inflammation and has been associated with prognosis in patients with advanced non–small cell lung cancer (NSCLC) (10,11).

In parallel, several publications suggest the existence of a lung–brain axis (12), indicating that diseases affecting the pulmonary system can lead to alterations in brain structure and function. Brain alterations have been observed in patients with lung cancer when compared with healthy controls, even before the initiation of therapy (13). Recent studies have also suggested a link between neuroinflammation and several prognostic factors, such as pathogenesis, cachexia (14), tumor growth (15), and systemic inflammation (16), calling for further investigations regarding the role of the brain function in patients with cancer.

The aim of this study was to evaluate the relationship between brain metabolic activity, measured using [^18^F]FDG PET, and survival in patients with advanced NSCLC.

## MATERIALS AND METHODS

This study was approved by the institutional review board of Institut Curie (DATA220207). Informed consent was provided by all patients. All related data were deidentified, collected, and stored in compliance with General Data Protection Regulation and the Declaration of Helsinki.

### Patients

This retrospective study included 455 patients with advanced NSCLC treated at Institut Curie between 2010 and 2023 who underwent baseline [^18^F]FDG PET/CT before any treatment. Patients were treated with the standard of care at the time of their management. Patients with no baseline [^18^F]FDG PET/CT before surgery or neoadjuvant therapy, a follow-up duration of less than 12 mo, missing clinical data, a PET/CT scan that did not fully include the brain in the axial field of view, and no lesion with an SUV of greater than 4 were excluded from the study. Clinical data, including age, sex, body mass index (BMI), smoking history, performance status (PS), stage, and treatment administered, were collected. Blood parameters, including albumin, C-reactive protein (CRP), monocytes, leukocytes, lymphocytes, neutrophils, and cortisol, were collected when available within 30 d of the PET scan and prior treatment initiation. The prognostic nutritional index (PNI), defined as albumin (grams per liter) + 5 × lymphocyte count (10^9^/liter), was calculated. Glycemic control was performed immediately before the PET scan. Patients’ history of depression was retrospectively collected from their medical records when the information was available.

**FIGURE 1.**
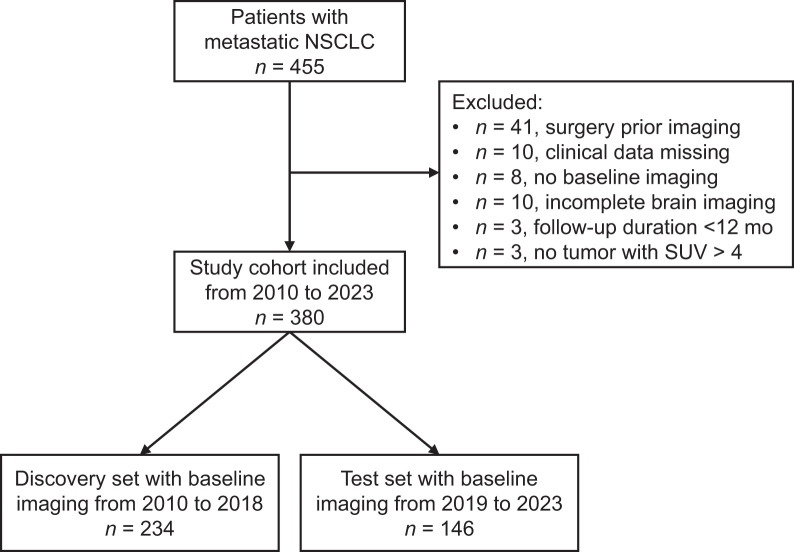
Flow diagram of study patients with advanced NSCLC.

The cohort was divided into 2 datasets: patients who underwent their PET/CT scan between January 2010 and December 2018 comprised the discovery set, whereas those scanned between January 2019 and December 2023 comprised the test set (Fig. 1).

OS was defined as the time between the baseline PET/CT scan acquisition and the date of the patient’s death or the last time the patient was known to be alive. Follow-up was determined from the date of the baseline PET/CT scan to the date of the last clinical consultation.

### Imaging and Image Analysis

[^18^F]FDG PET/CT scans were performed in various centers using different PET/CT systems and acquisition and reconstruction protocols (Supplemental Table 1, available at http://jnm.snmjournals.org). The mean blood glucose level before [^18^F]FDG PET/CT acquisition was 6.0 ± 1.4 mmol/L. An injected dose of 235 ± 75 MBq of [^18^F]FDG was administered 66 ± 11 min before image acquisition, with 1.8 ± 0.7 min per bed position. PET images were analyzed using SUV normalized to patient body weight.

All lesions were automatically segmented on PET images using LION version 0.14 (17). Lesion delineation was automatically refined by only including voxels with an SUV of 4 or greater. The whole brain was automatically delineated on the CT with TotalSegmentator version 2.0.5 (18), and the resulting volume of interest (VOI) was copied to the corresponding PET image (VOI_brain_). In addition, segmentation of 8 cerebral subregions, corresponding to the largest brain structures (lobes, ventricle, cerebellum, and brainstem), was performed.

For patients with brain metastases identified by any imaging modality (e.g., CT, MRI) at the time of the PET/CT scan or within 3 mo thereafter, we manually delineated brain lesions on the PET images, using the anatomic modality if necessary, and excluded these brain lesions from VOI_brain_, resulting in a volume corresponding to the whole brain without metastatic lesions (VOI_brain_noM_).

PET images were then resampled to a fixed voxel size of 2 × 2 × 2 mm^3^, and 5 tumor-related radiomic features were automatically extracted across all tumor lesions using LIFEX (version 26.3.8; http://www.lifexsoft.org) (19): D_max_, maximum SUV_max_, and total tumor SUV_mean_, TMTV, and total TLG (equal to tumor SUV_mean_ × TMTV). Cerebral metabolic activity was calculated as the mean [^18^F]FDG uptake within VOI_brain_, within the 8 cerebral subregions and also within VOI_brain_noM_, normalized by patient body weight (brain SUV_mean_ and brain SUV_mean_ without metastases, respectively) or normalized by lean body mass (brain SUL_mean_), using the following formulas (20):

$$\mathrm{Brain}\;{\mathrm{SUL}}_{\mathrm{mean}}\;(\mathrm{Female})=\frac{{\mathrm{SUV}}_{\mathrm{mean}}}{\mathrm{Weight}\;\left(g\right)}\times (1.07\times \mathrm{Weight}\;\left(\mathrm{kg}\right)-148\times \frac{{\mathrm{Weight}}^{2}\;\left(\mathrm{kg}\right)}{{\mathrm{Height}}^{2}\;\left(\mathrm{cm}\right)})\times 1000.$$ Eq. 1

$$\mathrm{Brain}\;{\mathrm{SUL}}_{\mathrm{mean}}\;\left(\mathrm{Male}\right)=\frac{{\mathrm{SUV}}_{\mathrm{mean}}}{\mathrm{Weight}\;\left(g\right)}\times (1.10\times \mathrm{Weight}\;(\mathrm{kg})-120\times \frac{{\mathrm{Weight}}^{2}\;\left(\mathrm{kg}\right)}{{\mathrm{Height}}^{2}\;\left(\mathrm{cm}\right)})\times 1000.$$ Eq. 2

### Statistical Analysis

#### Patient Characteristics

Baseline patient characteristics were analyzed using Wilcoxon tests for continuous variables and χ^2^ tests for categoric variables. Spearman correlation coefficients were calculated to assess the correlation between imaging features.

#### Evaluation of Automatic Segmentation

The reliability of the automatic segmentation performed by LION was assessed by comparison with the manual segmentation performed using an SUV threshold of 4 by an expert physicist under the supervision of a nuclear physician, in 100 randomly selected patients. The Dice score was calculated to assess agreement between expert and automated segmentation, and Bland–Altman analysis was performed to compare the 5 feature values as a function of the delineation method.

#### Survival Analysis

Associations between feature values and OS in the discovery set were studied using univariable Cox regression. All variables associated with OS at a *P* value of <0.05 in univariable analysis were included in a Cox multivariable model, without and with the brain [^18^F]FDG uptake (models 1 and 2, respectively). ANOVA was conducted to compare the 2 models. A risk score was generated from the multivariable models and then binarized by the median on the discovery set to distinguish between high- and low-risk patients by OS. We then applied the same models (same coefficients and same cutoff) to the test set. For both the discovery and test sets, the association of low-and high-risk groups with OS was evaluated using Kaplan–Meier curves with the log-rank test and a Cox proportional hazards regression model, including calculation of adjusted hazard ratios (HRs) with 95% CIs and Wald test *P* values.

#### Identification of Factors Affecting Cerebral Metabolic Activity

The influence of brain metastases on the prognostic value of cerebral metabolic activity was investigated by comparing the results obtained with VOI_brain_ and VOI_brain_noM_. The prognostic value of cerebral metabolic activity was also assessed in the subgroup of patients with advanced NSCLC without known brain metastases. The impact of the treatment on the prognostic value of cerebral metabolic activity was studied by categorizing patients by the treatment received after PET/CT (chemotherapy, radiotherapy, immunotherapy or targeted therapy). We used the Wilcoxon test and Spearman correlation coefficient to study the relationship between cerebral uptake and the presence of cerebral metastases, the technical PET/CT acquisition parameters, the value of biologic parameters, and patients’ depression history. We explored whether integrating cerebral metabolic activity with blood biomarkers could enhance prognostic stratification. For each prognostic biologic parameter, patients were stratified into 3 risk groups: low risk (0 risk factor), intermediate risk (1 risk factor), and high risk (2 risk factors). A *P* value of less than 0.05 was defined as statistically significant. All statistical analyses were conducted using R software version 4.2.2 (R Project for Statistical Computing).

Our study followed the criteria of the methodological radiomics score (21) and achieved a score of 84%, indicating high adherence to recommended radiomic research practices (Supplemental Fig. 1).

## RESULTS

### Patient Characteristics

In total, 380 patients (mean age, 65 ± 11 y) were included in this retrospective study (Fig. 1; Table 1). Our study cohort was divided into 2 groups by scan date: the discovery set (*n* = 234, 62%) and the test set (*n* = 146, 38%). Patients in the test set had a higher BMI (*P* = 0.004), 126 (86%) had a PS of 0 of 1 (*P* = 0.04), and 116 (79%) were treated with immunotherapy (*P* < 0.001). The test set also included a higher proportion of metastatic patients (99% had stage IV disease) compared with the discovery set (89%, *P* < 0.001).

**TABLE 1.** Demographic and Clinical Characteristics of Patients

Characteristic	Eligible cohort	Discovery set	Test set	*P* *
*n*	380	234	146	
Age at diagnosis (y)	65 ± 11	64 ± 11	66 ± 11	0.15
Sex				0.96
Female	162 (43)	99 (42)	63 (43)	
Male	218 (57)	135 (58)	83 (57)	
BMI (kg/m²)	23.8 ± 4.0	23.4 ± 4.0	24.4 ± 3.9	0.004
Histology				0.43
Adenocarcinoma	279 (73)	167 (71)	112 (77)	
Squamous cell carcinoma	52 (14)	36 (15)	16 (11)	
Other	49 (13)	31 (14)	18 (12)	
Stage				<0.001
III	29 (7)	27 (11)	2 (1)	
IVa	128 (34)	70 (30)	58 (40)	
IVb	223 (59)	137 (59)	86 (59)	
PS				0.04
0 or 1	307 (81)	181 (77)	126 (86)	
≥2	73 (19)	53 (23)	20 (14)	
Smoking history				0.57
Never	53 (14)	35 (15)	18 (12)	
Current or former	327 (86)	199 (85)	128 (88)	
Depressive disorders				0.84
Absence/not specified	362 (95)	222 (95)	140 (96)	
Presence	18 (5)	12 (5)	6 (4)	
Brain metastasis				0.31
Absence	293 (77)	185 (79)	108 (74)	
Presence	87 (23)	49 (21)	38 (26)	
Treatment received				
Chemotherapy	325 (86)	200 (85)	125 (86)	>0.99
Immunotherapy	222 (58)	106 (45)	116 (79)	<0.001
Radiotherapy	247 (65)	154 (66)	93 (64)	0.76
Targeted therapy	122 (32)	80 (34)	42 (29)	0.32
1-y survival				<0.001
Alive	233 (61)	131 (56)	102 (70)	
Dead	147 (39)	103 (44)	44 (30)	
Follow-up duration (mo)	24.6 ± 22.3	25.1 ± 25.5	23.7 ± 15.9	0.16

* *P* values from Wilcoxon and χ^2^ tests between discovery and test sets are shown.

Categoric data expressed as number, followed by percentage in parentheses; continuous data expressed as mean ± SD.

### Evaluation of Automated Segmentations

The tumor regions segmented by LION and by the experts were very similar, with an average Dice score of 0.90 ± 0.16 (range, 0.0–1.0) and no substantial differences in the derived radiomic feature values were found by Bland–Altman analyses (Supplemental Fig. 2). The prognostic performance of the 5 radiomic features was similar for both segmentation approaches, except for D_max_ (Supplemental Fig. 3). To ensure replicability, the results presented below were obtained using LION segmentation refined with a threshold of 4 SUV.

### Survival Analysis

Among all 380 patients, brain SUV_mean_ showed a significant but weak negative correlation with TMTV (*r* = −0.24) and total TLG (*r* = −0.21) (Supplemental Fig. 4). There was no significant correlation between brain SUV_mean_ and D_max_ (*r* = −0.09), total tumor SUV_mean_ (*r* = 0.03), and maximum SUV_max_ (*r* = 0.06) (*P* > 0.05). Total TLG was excluded from subsequent analyses because of its high correlation with TMTV (*r* = 0.98).

**FIGURE 2.**
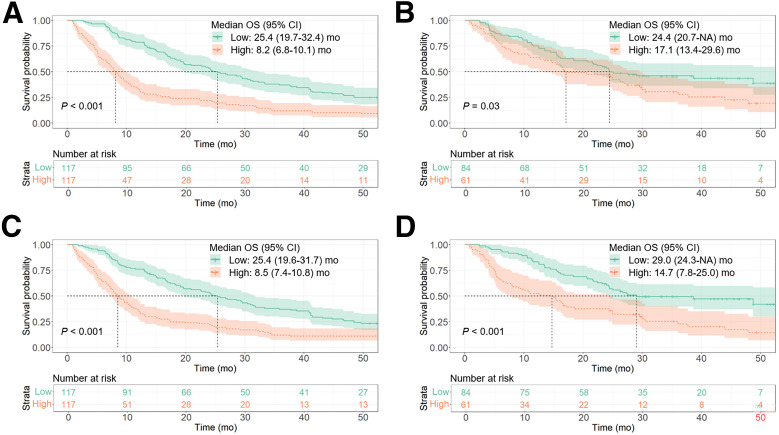
Kaplan–Meier curves for 2 multivariable models in discovery (*n* = 234) and test (*n* = 146) sets. Model 1 includes PS, smoking history, stage, D_max_, and TMTV for discovery (A) and test (B) sets. Model 2 includes features from model 1 and brain SUV_mean_ for discovery (C) and test (D) sets. Patients were stratified into risk groups using median value (1.56 for model 1 and 1.37 for model 2) derived from discovery set. Log-rank test *P* values are shown.

Kaplan–Meier curves for each feature are shown in Supplemental Figure 5 (continuous variable dichotomized by the median). In the discovery set, these univariable analyses identified 6 variables statistically associated with OS (Table 2): PS, smoking history, stage, D_max_, TMTV, and brain SUV_mean_. In the multivariable analysis without brain SUV_mean_ (model 1), all of these features were significantly associated with OS except D_max_. Using model 1, patients from the discovery set were divided into high- and low-risk groups on the basis of the score calculated from the multivariable Cox model dichotomized by the median value (1.56 [interquartile range, 0.5–42.7]). OS differed significantly between the 2 groups (*P* < 0.001) (Figs. 2A and 2B). Median OS was 25.4 mo (95% CI, 19.7–32.4 mo) and 8.2 mo (95% CI 6.8–10.1 mo) in the low-and high-risk groups, respectively.

**TABLE 2.** Cox Proportional Hazards Regression Analysis of Association of Clinical and Radiomic Features and Brain SUV_mean_ with OS in Discovery Set (*n* = 234)

			Multivariable analysis			
	Univariable analysis		Model 1*		Model 2^†^	
Variable	HR (95% CI)	*P* ^‡^	HR (95% CI)	*P* ^‡^	HR (95% CI)	*P* ^‡^
Age (y)	1.01 (1.00–1.02)	0.08				
Sex						
Female	—					
Male	1.19 (0.90–1.57)	0.21				
BMI (kg/m²)	0.99 (0.95–1.03)	0.56				
PS						
0 or 1	—		—		—	
≥2	2.20 (1.60–3.03)	<0.001	1.96 (1.41–2.71)	<0.001	1.88 (1.35–2.61)	<0.001
Smoking history						
Never	—		—		—	
Current or former	1.63 (1.10–2.42)	0.01	1.71 (1.13–2.59)	0.01	1.53 (1.00–2.35)	0.049
Stage						
III	0.91 (0.60–1.38)	0.64	1.01 (0.62–2.59)	0.97	0.97 (0.59–1.58)	0.89
IVa	0.62 (0.45–0.84)	0.002	0.65 (0.46–0.92)	0.01	0.65 (0.46–0.91)	0.01
IVb	—		—		—	
D_max_/10	1.07 (1.02–1.13)	0.009	1.03 (0.96–1.10)	0.35	1.03 (0.96–1.10)	0.42
TMTV/100	1.12 (1.06–1.19)	<0.001	1.12 (1.06–1.18)	<0.001	1.11 (1.04–1.17)	<0.001
Total TLG/100	1.02 (1.01–1.02)	<0.001				
Total tumor SUV_mean_	1.05 (0.99–1.12)	0.11				
Maximum SUV_max_	1.00 (1.00–1.01)	0.06				
Brain SUV_mean_	0.83 (0.76–0.92)	<0.001			0.89 (0.80–0.98)	0.02

* Includes all variables significantly associated with OS in univariable analysis (except brain SUV_mean_).

† Includes features from model 1 and brain SUV_mean_.

‡ Determined using Wald test.

When brain SUV_mean_ was used to build multivariable model 2, a significantly better patient stratification was observed when compared with model 1 (ANOVA, *P* = 0.02).

When model 1 was applied to the test set, OS significantly differed between the 2 risk groups (*P* = 0.03), with a median OS of 24.4 mo (95% CI 20.7–NA months) for 84 low-risk patients and 17.1 mo (95% CI 13.4–29.6 mo) for 61 high-risk patients, respectively. The addition of brain SUV_mean_ (model 2) improved the distinction between low- and high-risk patients in that test set (*P* < 0.001), with a difference in median OS of 14.3 mo between the 2 patient groups, compared with 7.3 mo with model 1. Very similar results were obtained when using brain SUL_mean_ (Supplemental Table 2; Supplemental Fig. 6).

**FIGURE 3.**
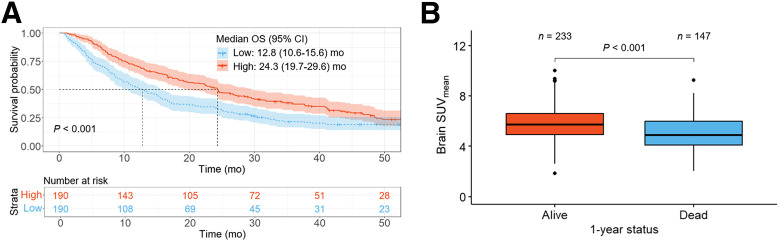
Relationship between brain SUV_mean_ and OS in patients with advanced NSCLC in eligible cohort (*n* = 380). (A) Kaplan–Meier curves (*P* value determined using log-rank test) for patients with NSCLC stratified into risk groups by median brain SUV_mean_ (median = 5.5). (B) Box plot representation of brain SUV_mean_ according to 1-y vital status in full cohort (*P* value determined using Wilcoxon test).

Patients who died within the first year of follow-up (61%) had a significantly lower median brain SUV_mean_ than did patients who were alive after 1 y (4.9 ± 1.4 vs. 5.7 ± 1.5, respectively; *P* < 0.001) (Fig. 3B; Supplemental Figs. 7B–7D). Similar results were found for brain SUL_mean_ (Supplemental Fig. 8). When stratifying patients by treatment received (Supplemental Fig. 9), the prognostic value of brain SUV_mean_ was confirmed for patients who received chemotherapy (86%), radiotherapy (65%), and immunotherapy (58%), but did not reach statistical significance in patients treated with targeted therapies (32%, *P* = 0.16). We also demonstrated that the combination of brain SUV_mean_ (or brain SUL_mean_) with TMTV enables the identification of 3 distinct risk groups associated with patient survival (Supplemental Fig. 10).

### Identification of Factors Affecting Cerebral Metabolic Activity

Among the 87 patients with brain metastases, only 29 (33%) had either metastases or perilesional edema visible on PET images. Low cerebral metabolic activity was significantly associated with poor OS (*P* < 0.001), regardless of the use of VOI_brain_ or VOI_brain_noM_ (Fig. 3A; Supplemental Figs. 7A–7C). When the whole-brain was automatically segmented by TotalSegmentator (VOI_brain_), we observed no significant difference in brain SUV_mean_ distribution between patients with brain metastases and those without (*P* = 0.68). Low [^18^F]FDG uptake in the brainstem, cerebellum, and each of the 5 lobes was significantly associated with poor OS (*P* < 0.04; Supplemental Fig. 11), but no more than the mean [^18^F]FDG uptake over the whole brain (brain SUV_mean_).

Brain SUV_mean_ was significantly but weakly correlated with BMI (*r* = 0.26; Supplemental Fig. 12) and negatively correlated with age (*r* = −0.20). Moreover, brain SUV_mean_ was significantly lower in current smokers than in nonsmokers (*P* = 0.002) as well as in those with a PS of 2 or greater compared with those with a PS of 0 or 1 (*P* < 0.001). There was no significant correlation between brain SUV_mean_ and technical parameters influencing uptake, namely the injected dose, time from injection to acquisition, and acquisition duration (Fig. Supplemental 13A).

Regarding biologic data, blood biomarkers were available for 110 patients (29%) (Supplemental Figs. 13B and 14). Brain SUV_mean_ was positively correlated with albumin (*r* = 0.31), lymphocytes (*r* = 0.15), and PNI (*r* = 0.35) and negatively correlated with blood glucose levels (*r* = −0.43), neutrophils (*r* = −0.38), CRP (*r* = −0.37), leukocytes (*r* = −0.37), and monocytes (*r* = −0.32). The correlation between brain SUV_mean_ and cortisol was not significant (*P* = 0.26). Kaplan–Meier curves of each blood parameter, enabling patient stratification into 2 groups of risk on the basis of normal biologic values, are shown in Supplemental Figures 15A–15F. Patients who died within the first year (46/110, 43%) had higher levels of CRP, leukocytes, monocytes, and neutrophils and lower levels of lymphocytes compared with patients who were still alive after 1 y (Supplemental Figs. 15G–15L). However, there were no significant differences in albumin levels between risk groups (*P* = 0.30). Blood glucose levels were significantly associated with survival (*P* < 0.001), with higher values observed in patients who died within the first year (*P* = 0.02) (Supplemental Fig. 16). Kaplan–Meier curves showed significantly different OS among patients stratified by 0, 1, or 2 risk factors, defined by the association of brain SUV_mean_ with blood biomarkers (*P* ≤ 0.04) (Supplemental Figs. 17 and 18), except for cortisol (*P* = 0.42, data available for 42 patients). Low PNI was significantly associated with poorer survival (*P* = 0.02), and its combination with brain SUV_mean_ identified patients with a poorer prognosis than those with low PNI alone (PNI < 46.9 and brain SUV_mean_ < 5.5) (Supplemental Fig. 19).

Finally, patients with known depressive disorders (*n* = 18) had lower median brain [^18^F]FDG uptake (brain SUV_mean_, 4.9 ± 1.3; brain SUL_mean_, 3.9 ± 1.0) than those without these disorders (brain SUV_mean_, 5.5 ± 1.5; brain SUL_mean_, 4.2 ± 1.1), but the difference was not statistically significant (Supplemental Fig. 20). The presence of depressive disorders was not significantly associated with OS.

**FIGURE 4.**
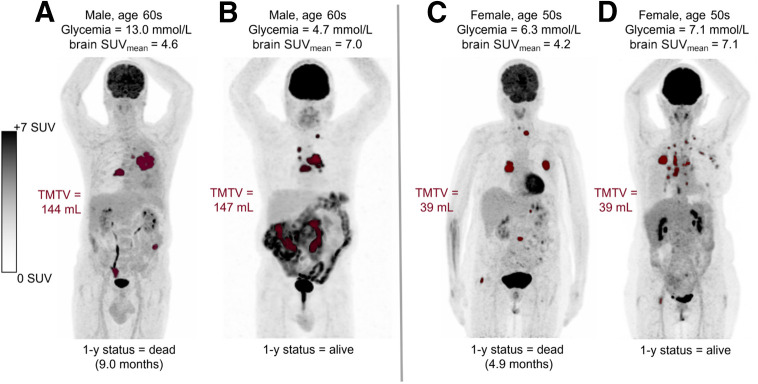
Examples of baseline [^18^F]FDG PET images of 4 patients with metastatic NSCLC. Primary tumor and metastatic lesions are delineated in red. TMTV, brain SUV_mean_, and 1-y survival status are shown.

Figure 4 shows patients with similar clinical and radiomic features but differing brain SUV_mean_ and their associated survival.

## DISCUSSION

Recent studies have suggested that [^18^F]FDG metabolic activity measured across various organs and tissues outside the tumor site could provide valuable information for predicting patient outcomes in lung cancer (8,9). We found that brain FDG uptake was an independent prognostic factor in both univariable and multivariable analyses and complemented radiomic features reflecting tumor invasion. Brain FDG uptake, combined with clinical and tumor-related radiomic features measured on baseline [^18^F]FDG PET/CT scans, stratified patients with advanced NSCLC into low- and high-risk groups according to their OS (*P* < 0.001). Low brain FDG uptake was significantly associated with a poor prognosis, regardless of the treatment received after PET/CT, except for targeted therapy (122 patients).

When studying the metabolism of healthy organs, the “tumor sink effect” should be considered. In this phenomenon, increased tumor burden, whether attributable to lesion size, number, or aggressiveness, reduces the availability of the tracer to healthy tissues, thereby reducing their uptake (22). We found only a weak correlation between brain [^18^F]FDG uptake and TMTV (*r* = −0.24; Supplemental Fig. 4) and showed that these 2 parameters were complementary to predict OS in NSCLC (Supplemental Fig. 10), demonstrating that the prognostic value of brain SUV_mean_ cannot be explained by the tumor sink effect.

Several assumptions can be made to explain our observations. One is a possible relationship between brain metabolism and systemic inflammation. A recent study demonstrated the existence of a circuit capable of detecting inflammation in the blood and regulating the antiinflammatory response through the brain, revealing a bidirectional communication between the brain and the immune system (23). Inflammation is one of the inevitable consequences of tumorigenesis, leading to the recruitment of inflammatory cells, such as monocytes, to tumor sites via the bloodstream (24). In our study, we observed moderate but significant negative correlations between brain SUV_mean_ and CRP or monocytes. The combination of brain SUV_mean_ with these features stratified patients into 3 groups with significantly different OS (*P* ≤ 0.002; Supplemental Fig. 17), supporting their complementary prognostic value. Overall, these findings suggest that cerebral glucose metabolism measured on PET images may reflect mechanisms involved in regulating the antiinflammatory response, which is associated with immune cell counts and inflammatory response proteins, such as CRP.

Another possible explanation could be a correlation between cerebral [^18^F]FDG uptake and the patient’s functional status, encompassing physical or cognitive components. Our analysis demonstrated that cerebral [^18^F]FDG uptake is weakly but significantly positively or negatively correlated with age, BMI, smoking status, PS, serum albumin levels, and blood glucose levels, consistent with previous studies that have individually reported associations between some of these parameters and cerebral uptake (25,26). Taken together, these findings suggest that brain SUV_mean_ may reflect the patient’s overall functional and nutritional statuses, which are known to be associated with clinical prognosis (27,28). Other studies using [^18^F]FDG PET/CT have reported a correlation between decreased cerebral glucose metabolism and psychologic conditions in patients with lung cancer (29). Patients with cancer face a higher risk of depression, with a prevalence influenced by sex, age, and cancer type (30). It has been reported that patients with lung cancer have lower brain [^18^F]FDG uptake than do patients with other cancer types and a higher incidence of depression and anxiety, particularly patients with metastases (29). In our study, patients with documented depressive disorders (5%) had lower brain [^18^F]FDG uptake than those without such disorders (95%), although the difference was not statistically significant (*P* = 0.33). Moreover, several studies have reported a link between patients with depressive syndromes and blood biomarkers associated with systemic inflammation (31–33) and between nutritional status and mental condition (34). In addition, advanced arteriosclerosis of the carotid and cerebral vessels could influence the availability of [^18^F]FDG to the brain and explain a low brain SUV_mean_. This hypothesis cannot be excluded, as 86% of patients in our cohort were smokers; however, this could not be tested because of the lack of information regarding their vascular status. Further studies are needed to elucidate the main biologic mechanisms reflected by brain metabolism.

Furthermore, we investigated whether [^18^F]FDG uptake in individual brain structures provided additional prognostic information; however, regional brain metabolism of 8 major regions did not outperform average brain uptake for predicting prognosis (Fig. 3; Supplemental Fig. 11).

This study had several limitations. One such limitation was the significant difference between the composition of the discovery and test sets. We divided the cohort by PET scan date, introducing bias in the treatment selection and OS. Indeed, patients in the test cohort had access to more innovative treatments, such as immunotherapy. Yet, the models could stratify patients in the test set without adjusting the cutoff values. Because of the heterogeneity of treatments and the limited number of patients in most subgroups, the effect of combined therapies on cerebral metabolism could only be assessed in the subgroup receiving chemotherapy, radiotherapy, and immunotherapy—the largest subgroup with 108 patients—where the prognostic value of cerebral metabolism for OS remained significant (data not shown). Another limitation was the incomplete availability of biologic data, which may have limited the statistical power of some analyses. Finally, we retrospectively collected information on depression from patient medical records, which is incomplete and imperfect, as patients may not necessarily report a depressive disorder or may be unaware of it. To investigate the link between brain SUV_mean_, patient’s functional status and survival, we will need to prospectively collect a score measuring depression at the time of baseline [^18^F]FDG PET/CT scan, such as the Hamilton Depression Rating Scale (35), possibly use connected devices to measure the patient’s physical condition and activity, and investigate the impact of concomitant medications. Our results warrant further studies to investigate the relationship between cerebral [^18^F]FDG uptake and the patient’s functional status in oncology. Depending on the results of these investigations, brain SUV_mean_ could potentially be used to identify patients who should be prioritized for supportive care interventions, such as psychologic support, nutritional counseling, tailored physical activity programs, or the prescription of a comprehensive cardiovascular evaluation.

## CONCLUSION

Low brain [^18^F]FDG uptake was associated with an increased risk of mortality in patients with advanced NSCLC. Accounting for brain [^18^F]FDG uptake in addition to clinical and tumor-related radiomic features improved OS prediction compared with relying only on clinical and tumor-related radiomic features. Further studies investigating the role of brain [^18^F]FDG uptake as a prognostic biomarker for OS and its association with systemic inflammation and the patient’s functional status are warranted.

## DISCLOSURE

This work was supported by the French National Research Agency (ANR-22-CE45-0001 NEMO-PET). Nicolas Girard reports a consulting or advisory role for Abbvie, Amgen, AstraZeneca, BeiGene, Bristol-Myers Squibb, Daiichi Sankyo/AstraZeneca, Gilead Sciences, Ipsen, Janssen, LEO Pharma, Lilly, MSD, Novartis, Pfizer, Roche, Sanofi, and Takeda Pharmaceuticals. Lalith Sundar and Thomas Beyer are cofounders of Zenta GmbH. No other potential conflict of interest relevant to this article was reported.

## Notes

KEY POINTS **QUESTION:** Could brain metabolic activity measured by [^18^F]FDG PET/CT serve as an independent prognostic factor for survival in patients with advanced non–small cell lung cancer? **PERTINENT FINDINGS:** Low pretreatment brain [^18^F]FDG uptake was associated with an increased risk of mortality in patients with advanced NSCLC. Brain [^18^F]FDG uptake predicts survival regardless of the presence of brain metastases; it is independent of tumor volume and metabolic activity and is significantly correlated, yet complementary, to several clinical and blood biomarkers, including CRP. **IMPLICATIONS FOR PATIENT CARE:** Brain [^18^F]FDG uptake is a prognostic biomarker for survival in patients with advanced NSCLC and may help identify patients who would benefit most from prioritized supportive care interventions.

## References

[1] Bray F Laversanne M Sung H. Global cancer statistics 2022: GLOBOCAN estimates of incidence and mortality worldwide for 36 cancers in 185 countries. CA Cancer J Clin. 2024;74:229–263. 38572751 10.3322/caac.21834

[2] Chansky K Detterbeck FC Nicholson AG.; IASLC Staging and Prognostic Factors Committee, Advisory Boards, and Participating Institutions. The IASLC Lung Cancer Staging Project: external validation of the revision of the TNM stage groupings in the eighth edition of the TNM Classification of Lung Cancer. J Thorac Oncol. 2017;12:1109–1121. 28461257 10.1016/j.jtho.2017.04.011

[3] Belaroussi Y Bouteiller F Bellera C. Survival outcomes of patients with metastatic non-small cell lung cancer receiving chemotherapy or immunotherapy as first-line in a real-life setting. Sci Rep. 2023;13:9584. 37311845 10.1038/s41598-023-36623-1 PMC10264352

[4] Planchard D Popat S Kerr K.; ESMO Guidelines Committee. Metastatic non-small cell lung cancer: ESMO clinical practice guidelines for diagnosis, treatment and follow-up. Ann Oncol. 2018;29(suppl 4):iv192–iv237. 30285222 10.1093/annonc/mdy275

[5] Tricarico P Chardin D Martin N. Total metabolic tumor volume on ^18^F-FDG PET/CT is a game-changer for patients with metastatic lung cancer treated with immunotherapy. J Immunother Cancer. 2024;12:e007628. 38649279 10.1136/jitc-2023-007628 PMC11043703

[6] Huang M Zou Y Wang W Li Q Tian R. The role of baseline ^18^F-FDG PET/CT for survival prognosis in NSCLC patients undergoing immunotherapy: a systematic review and meta-analysis. Ther Adv Med Oncol. 2024;16:17588359241293364. 39502406 10.1177/17588359241293364 PMC11536524

[7] Pellegrino S Fonti R Morra R. Prognostic value of tumor dissemination (Dmax) derived from basal ^18^F-FDG positron emission tomography/computed tomography in patients with advanced non-small-cell lung cancer. Biomedicines. 2025;13:477. 40002890 10.3390/biomedicines13020477 PMC11853205

[8] Salimi Y Hajianfar G Mansouri Z. Organomics: a concept reflecting the importance of PET/CT healthy organ radiomics in non-small cell lung cancer prognosis prediction using machine learning. Clin Nucl Med. 2024;49:899–908. 39192505 10.1097/RLU.0000000000005400

[9] Zhang Y Tan W Zheng Z Wang J Xing L Sun X. Body composition and radiomics from ^18^F-FDG PET/CT together help predict prognosis for patients with stage IV non-small cell lung cancer. J Comput Assist Tomogr. 2023;47:906–912. 37948365 10.1097/RCT.0000000000001496

[10] Lee JW Choi JS Lyu J Lee SM. Prognostic significance of ^18^F-fluorodeoxyglucose uptake of bone marrow measured on positron emission tomography in patients with small cell lung cancer. Lung Cancer. 2018;118:41–47. 29572001 10.1016/j.lungcan.2018.01.020

[11] Seban RD Assié JB Giroux-Leprieur E. Prognostic value of inflammatory response biomarkers using peripheral blood and [^18^F]-FDG PET/CT in advanced NSCLC patients treated with first-line chemo- or immunotherapy. Lung Cancer. 2021;159:45–55. 34311344 10.1016/j.lungcan.2021.06.024

[12] He M Liu Y Guan Z Li C Zhang Z. Neuroimaging insights into lung disease-related brain changes: from structure to function. Front Aging Neurosci. 2025;17:1550319. 40051465 10.3389/fnagi.2025.1550319 PMC11882867

[13] Zhang W Ning N Li X. Changes of brain glucose metabolism in the pretreatment patients with non-small cell lung cancer: a retrospective PET/CT study. PLoS One. 2016;11:e0161325. 27529342 10.1371/journal.pone.0161325 PMC4987062

[14] Molfino A Gioia G Rossi Fanelli F Laviano A. Contribution of neuroinflammation to the pathogenesis of cancer cachexia. Mediators Inflamm. 2015;2015:801685. 26504362 10.1155/2015/801685 PMC4609516

[15] Norden DM Bicer S Clark Y. Tumor growth increases neuroinflammation, fatigue and depressive-like behavior prior to alterations in muscle function. Brain Behav Immun. 2015;43:76–85. 25102452 10.1016/j.bbi.2014.07.013 PMC4258420

[16] Cunningham C. Microglia and neurodegeneration: the role of systemic inflammation. Glia. 2013;61:71–90. 22674585 10.1002/glia.22350

[17] Pires M Ferrara D Beyer T Shiyam Sundar LK. Fully automated FDG and PSMA lesion segmentation in PET imaging via deep learning [abstract]. Eur J Nucl Med Mol Imaging. 2024;51(suppl 1).

[18] Wasserthal J Breit HC Meyer MT. TotalSegmentator: robust segmentation of 104 anatomic structures in CT images. Radiol Artif Intell. 2023;5:e230024. 37795137 10.1148/ryai.230024 PMC10546353

[19] Nioche C Orlhac F Boughdad S. LIFEx: a freeware for radiomic feature calculation in multimodality imaging to accelerate advances in the characterization of tumor heterogeneity. Cancer Res. 2018;78:4786–4789. 29959149 10.1158/0008-5472.CAN-18-0125

[20] Sugawara Y Zasadny KR Neuhoff AW Wahl RL. Reevaluation of the standardized uptake value for FDG: variations with body weight and methods for correction. Radiology. 1999;213:521–525. 10551235 10.1148/radiology.213.2.r99nv37521

[21] Kocak B Akinci D’Antonoli T Mercaldo N. METhodological RadiomICs Score (METRICS): a quality scoring tool for radiomics research endorsed by EuSoMII. Insights Imaging. 2024;15:8. 38228979 10.1186/s13244-023-01572-w PMC10792137

[22] Beauregard JM Hofman MS Kong G Hicks RJ. The tumour sink effect on the biodistribution of ^68^Ga-DOTA-octreotate: implications for peptide receptor radionuclide therapy. Eur J Nucl Med Mol Imaging. 2012;39:50–56. 21932117 10.1007/s00259-011-1937-3

[23] Jagot F Gaston-Breton R Choi AJ. The parabrachial nucleus elicits a vigorous corticosterone feedback response to the pro-inflammatory cytokine IL-1β. Neuron. 2023;111:2367–2382.e6. 37279750 10.1016/j.neuron.2023.05.009

[24] Chen X Li Y Xia H Chen YH. Monocytes in tumorigenesis and tumor immunotherapy. Cells. 2023;12:1673. 37443711 10.3390/cells12131673 PMC10340267

[25] Yoshizawa H Gazes Y Stern Y Miyata Y Uchiyama S. Characterizing the normative profile of ^18^F-FDG PET brain imaging: sex difference, aging effect, and cognitive reserve. Psychiatry Res. 2014;221:78–85. 24262800 10.1016/j.pscychresns.2013.10.009

[26] Sharma P Chatterjee P Alvarado LA Dwivedi AK. Standardized uptake value of normal organs on routine clinical [^18^F]FDG PET/CT: impact of tumor metabolism and patient-related factors. Nucl Med Rev Cent East Eur. 2023;26:1–10. 36286203 10.5603/NMR.a2022.0036

[27] Jones LW Hornsby WE Goetzinger A. Prognostic significance of functional capacity and exercise behavior in patients with metastatic non-small cell lung cancer. Lung Cancer. 2012;76:248–252. 22112290 10.1016/j.lungcan.2011.10.009 PMC3615546

[28] Zhang Q Bao J Zhu ZY Jin MX. Prognostic nutritional index as a prognostic factor in lung cancer patients receiving chemotherapy: a systematic review and meta-analysis. Eur Rev Med Pharmacol Sci. 2021;25:5636–5652. 34604956 10.26355/eurrev_202109_26783

[29] Yang X Yang G Wang R. Brain glucose metabolism on [^18^F]-FDG PET/CT: a dynamic biomarker predicting depression and anxiety in cancer patients. Front Oncol. 2023;13:1098943. 37305568 10.3389/fonc.2023.1098943 PMC10248443

[30] Linden W Vodermaier A Mackenzie R Greig D. Anxiety and depression after cancer diagnosis: prevalence rates by cancer type, gender, and age. J Affect Disord. 2012;141:343–351. 22727334 10.1016/j.jad.2012.03.025

[31] McFarland DC Jutagir DR Miller AH Breitbart W Nelson C Rosenfeld B. Tumor mutation burden and depression in lung cancer: association with inflammation. J Natl Compr Canc Netw. 2020;18:434–442. 32259781 10.6004/jnccn.2019.7374 PMC7327956

[32] Andersen BL Myers J Blevins T. Depression in association with neutrophil-to-lymphocyte, platelet-to-lymphocyte, and advanced lung cancer inflammation index biomarkers predicting lung cancer survival. PLoS One. 2023;18:e0282206. 36827396 10.1371/journal.pone.0282206 PMC9956881

[33] Ninla-Aesong P Kietdumrongwong P Neupane SP. Relative value of novel systemic immune-inflammatory indices and classical hematological parameters in predicting depression, suicide attempts and treatment response. Sci Rep. 2024;14:19018. 39152198 10.1038/s41598-024-70097-z PMC11329510

[34] Chabowski M Polański J Jankowska-Polańska B Janczak D Rosińczuk J. Is nutritional status associated with the level of anxiety, depression and pain in patients with lung cancer? J Thorac Dis. 2018;10:2303–2310. 29850135 10.21037/jtd.2018.03.108 PMC5949507

[35] Hamilton M. A rating scale for depression. J Neurol Neurosurg Psychiatry. 1960;23:56–62. 14399272 10.1136/jnnp.23.1.56 PMC495331

